# Acquisition of non-canonical word orders in Mandarin Chinese

**DOI:** 10.3389/fpsyg.2023.1006148

**Published:** 2023-04-17

**Authors:** Yue Ji, Li Sheng, Li Zheng

**Affiliations:** ^1^Department of English, School of Foreign Languages, Beijing Institute of Technology, Beijing, China; ^2^Department of Chinese and Bilingual Studies, The Hong Kong Polytechnic University, Kowloon, Hong Kong SAR, China; ^3^College of Education Science, Nanjing Normal University, Nanjing, China

**Keywords:** *ba*-construction, *bei*-construction, word order, language acquisition, Mandarin

## Abstract

To better understand Mandarin-speaking children’s acquisition of non-canonical word orders, we tested comprehension and production of Mandarin non-canonical active *ba*-construction and passive *bei*-construction, in comparison with canonical active SVO sentences among 180 children between three and 6 years of age. Our results showed that children had more difficulties with *bei*-construction compared to SVO sentences in both comprehension and production, but early problems of *ba*-construction only lied in production. We discussed these patterns in connection with two accounts of language acquisition which attribute language development to the maturation of grammar or to the exposure to the input, respectively.

## 1. Introduction

One of the most basic tasks for language learners is to correctly understand and express who does what to whom, which is typically encoded in an active sentence with the canonical SVO word order in Mandarin like (1a). Meanwhile, Mandarin has non-canonical *ba*-construction (S-*ba*-O-V) and passive *bei*-construction (O-*bei*-S-V) as shown in (1b) and (1c).

(1) a. laohu yao-lee ’yu.

tiger bite-PFV crocodile.

‘The tiger bit the crocodile.’

b. laohu **ba** e’yuyao-le.

tiger BA crocodile bite-PFV.

‘The tiger bit the crocodile.’

c. laohu **bei** e’yuyao-le.

tiger BEI crocodile bite-PFV.

‘The tiger was bitten by the crocodile.’

How does a Mandarin-speaking child learn to identify the agent and the patient in these different structures? Theoretical accounts for this issue at the syntax-semantics interface mainly fall into two camps. The maturation approach couched in generative grammar proposes that representations of underlying syntactic structures are innate, but the derivations of some syntactic constructions are not fully represented in child grammar until a certain age (e.g., Borer and Wexler (1987)’s A-Chain Deficit Hypothesis; Chomsky, 1995). By contrast, the usage-based approach suggests that the syntactic constructions gradually build up as children get more exposure to instances of these constructions in the input (e.g., Tomasello, 2003; Goldberg, 2006; see Abbot-Smith and Tomassello, 2016 for a review). The *ba-* and *bei-*constructions in Mandarin involve different syntactic movements and are used with different frequencies, thus they can serve as a good test case for examining the two accounts, and provide new insights into the nature of early syntactic development.

In the present study, we explore Mandarin-speaking children’s acquisition of *ba*- and *bei-*constructions compared to the canonical SVO sentences. In the remainder of the Introduction, we will briefly introduce the syntactic and semantic properties of *ba* and *bei* sentences, and discuss the implications for acquisition with a review of relevant developmental works. We will end this section by raising the research questions of our study.

In both *ba-* and *bei*-constructions, *ba* and *bei* appear between two noun phrases and indicate the thematic roles of the two arguments (Li and Thompson, 1981). In *ba-*construction like (1b), the noun phrase after *ba* is typically the patient; and the construction implies that the patient is “being affected, dealt with or disposed of” (see Sybesma, 1999, p. 132, for a summary). In *bei-*construction like (1c), the noun phrase after *bei* is typically the agent while the noun phrase in the surface subject position before *bei* is the patient. The *bei*-construction highlights the patient and its affectedness (Li, 1990; Sun, 1991).

In the generative framework*, ba* is widely regarded as a light verb that assigns an Accusative Case to its object (Bender, 2000; Tian, 2003; Huang et al., 2009). The derivation of *ba*-construction involves A-movement in which the post-verb patient argument raises to the preverbal position and receives Case from *ba* as shown below (Huang et al., 2009).

(2) (=1b) laohu ba e’yu yao-le

tiger BA crocodile bite-PFV.

The derivation of the passive *bei*-construction is more complex and has been under debate. Here we follow Huang’s (1999) approach treating *bei* as a verb that takes a clausal complement (IP) (see more elaboration in Huang, 2013 and a similar proposal in Bruening and Tran, 2015). The object of the embedded clause is a null operator (OP) and it moves to the specifier position of the embedded clause ([Spec, IP]), then it forms a relation of predication with the matrix subject as shown in (3). Semantically, *bei*-sentences like (3) express that the matrix subject (*laohu* “tiger” in (3)) ends up with the property of being an x such that the embedded subject (*e’yu* “crocodile”) acts upon (*yao* “bite”) x (Huang et al., 2009).

(3) (=1c) laohu bei e’ yuyao-le.

tiger BEI crocodile bite-PFV.

The *ba-* and *bei*-constructions have often been discussed together since they are closely related variations of the SVO order: in general, the subject of the *ba*-construction takes an agent role and corresponds to the noun phrase appearing after *bei* while the object of *ba*, taking a patient role, would surface as the matrix subject of the *bei*-construction. But these two constructions differ in an important way: the noun phrase after *ba* cannot be omitted as shown in (4); the noun phrase after *bei* can be omitted and (5) is called a short passive compared to the long passive in (3). Unlike English where short and long passives have the same syntactic structure and the *by-*phrase is optional, short passives in Mandarin undergo a different syntactic movement from long passives (Huang et al., 2009). Our study is focused on long passives. We are interested in how children interpret and express the two event roles (i.e., agent and patient) explicitly in different constructions.

(4) * laohu bayao-le.

tiger BAbite-PFV.

Intended meaning: ‘The tiger bit someone.’

(5) laohu bei yao-le.

tiger BEI bite-PFV.

‘The tiger was bitten (by someone).’

Both *ba-* and *bei*-constructions are non-canonical as the thematic roles and syntactic positions are not canonically mapped in syntactic derivation. Moreover, these two constructions are much less frequent compared to SVO sentences. To evaluate the availability of both constructions in children’s input, we examined child-directed speech in Zhou’s (2001) corpus, which includes cross-sectional data of 40 mother–child pairs. In this corpus, the Mandarin-speaking children fall into four age groups—14, 20, 26 and 32 months—with 10 children in each group; thus the parent speech could reflect what children hear when their language is growing from the two-word stage to full sentences. The context for the interaction between a mother and her child was a semi-structured play scenario, which is common and representative of everyday communication. Among the total 7,101 parent utterances after removing repetitions and unintelligible utterances, 4,040 utterances were SVO sentences, 332 were *ba* sentences, and only two were *bei* sentences, including one long passive and one short passive (the rest were interjections or one-word utterances). This suggests that *bei*-construction is extremely rare in the input. Apart from syntactic complexity, *bei*-construction has the semantic connotation that the patient argument has undergone some adverse influence from the action of the agent argument (e.g., Shi, 2005), which may contribute to the extremely low frequency of the construction in everyday parent–child interactions.

According to the maturation account, the formation of *ba*-construction and *bei*-construction undergoes syntactic movements. However, the former involves A-movement which occurs within the vP, while the latter involves operator movement in which the null operator moves out of the vP to the edge of IP. Would the different types of movement have an effect on learning the two constructions? In the Minimalist framework, Chomsky (2001) proposed the Phase Impenetrability Condition (PIC) in (6a), under which the object of a verb cannot move out of the vP phase unless *v* is “defective” (i.e., it does not assign an external argument as in passives). Based on PIC, Wexler (2004, 2007) in his account for English-speaking children’s difficulties with passives proposed that unlike adults, young children lack knowledge about “defective” *v* (see the Universal Phase Requirement, UPR in (6b)). In other words, in a premature grammar, all vPs are barriers that prevent the object of a verb to move further. Thus passive sentences are misunderstood as active sentences by young children until the maturation of their grammar.

(6) a. PIC: When working at a phase, the edge (the head and any specifiers) of the next lower phase is available for analysis, but nothing lower than the edge. In particular the complement is not available.

b. UPR: for immature children until around age five, *v* always defines a phase, whether or not *v* is defective.

If PIC is innate and UPR is universal (as assumed by the maturation approach), then they would predict that *ba*-construction is available to Mandarin-speaking children from the beginning since the construction only involves a vP-internal movement. In comparison, acquisition of *bei*-construction which involves long-distance movement out of vP is delayed until the maturation of knowledge about “defective” *v* and young children may misinterpret *bei*-sentences as active sentences.

Previous studies have shown Mandarin-speaking children’s difficulties with *bei*-construction. Using act-out experiments, Chang (1986) showed that even 5-year-olds had non-adult-like comprehension of *bei*-construction (e.g., they misunderstood *bei*-sentences as active sentences). The acquisition of *ba*-construction has also attracted some attention and has often been compared with the acquisition of passives. Using grammaticality judgment and sentence correction tasks, Gong (2007) tested *ba-* and *bei*-constructions in 4- and 5-year-olds. The results revealed a clear development from age 4 to age 5, but even 5-year-olds made considerable mistakes in both constructions; particularly, the performance on *ba*-construction seemed not to be better than that on *bei*-construction. Liu and Ning (2009) compared the acquisition of unaccusatives, *ba-*construction, and *bei*-construction in children from age 2 to age 6. In that study, worse performance on *bei*-construction compared to the other two was detected in both comprehension and elicited-production tasks. Recent works adopted online measurements to investigate children’s processing of *ba-* and *bei-*constructions. Huang et al. (2013) used the visual-world paradigm to examine how 5-year-olds (and adults) assigned thematic roles in sentence comprehension. Their results suggest that passives may involve re-assignment of agent and patient roles, which would lead to processing difficulties and comprehension errors. In comparison, Zhou and Ma (2018) used the same paradigm and found that both 3-year-olds and 5-year-olds could use *ba* and *bei* as cues to correctly assign thematic roles. In sum, prior research has adopted a variety of methods exploring the comprehension and production of *ba-* and *bei-*constructions in preschool children, but results concerning whether passives are more difficult and acquired later than *ba-*construction are mixed. Moreover, most of prior studies have not systematically compared *ba-* and *bei*-constructions against the canonical SVO sentences in children’s acquisition, and thus it is impossible to examine the effect of syntactic movement in the generative framework. In addition, it is not clear what errors children made in previous tasks.

Apart from the syntactic derivation, *ba-* and *bei*-constructions differ in input frequency. The usage-based account of language acquisition argues that frequency plays an important role in children’s learning of a specific construction. For instance, the early difficulties with passives in English-speaking children are attributed to a lack of exposure to passive sentences, which have a low frequency in adult speech (Harris and Flora, 1982; Gordon and Chafetz, 1990; Brooks and Tomasello, 1999). This account predicts that Mandarin-speaking children would acquire SVO sentences first, and then the *ba*-construction, while *bei*-construction would be learned even later due to the extreme poverty of the input. Previous naturalistic studies on Mandarin-speaking children’s early production confirmed the prediction to some extent. *Ba* sentences emerged around age 2 after the appearance of SVO sentences (Yang and Xiao, 2008). 3-year-old children could produce *ba* sentences with novel verbs (Hsu, 2014). By 4 and a half years, children could use more complex *ba*-constructions (e.g., using resultative verb compounds instead of single verbs) and their production became adult-like (Li et al., 1990; Li, 1995; Deng et al., 2018). By contrast, *bei*-construction did not appear until 2 and a half years and more importantly, early *bei*-sentences were short passives, the derivation of which does not involve null-operator movement and thus is structurally simpler (Zhou et al., 1992; Zhou, 1997). In addition, children seem to be sensitive to the aspectual properties of *ba* and *bei* from early on: both *ba* and *bei* sentences occur more frequently in perfective than imperfective aspect in child speech (Deng et al., 2018). However, naturalistic data mainly show the production of different sentence structures in young children. What children can produce might be different from what they habitually produce in everyday life. Moreover, it is impossible to assess what children avoid in their utterances. Therefore, children’s linguistic competence may not be fully reflected from the naturalistic data.

To sum up, the SVO word order is canonical and unmarked in Mandarin. *Ba*-construction and *bei*-construction are non-canonical word orders and in both constructions, the patient argument is not in the post-verbal object position. Semantically, both constructions highlight the affectedness of the patient argument. Despite their similarities, the two constructions involve two different types of syntactic movement and differ in their frequencies in child-directed speech.

The present study measures how well children (3;6-6;5) comprehend and produce *ba* and *bei* sentences compared to the canonical SVO sentences. First, the results are expected to reveal whether the two constructions have similar developmental patterns in preschool children given the mixed findings in prior research. Second, *ba-* and *bei-*constructions can be taken as a test case to evaluate the maturation account and the usage-based account of language acquisition. Under the maturation account, we would expect similar performance between *ba* and SVO sentences but worse performance on *bei* sentences. By contrast, under the usage-based account, we would expect performance to be best on SVO sentences, poorer on *ba*-construction, and the poorest on *bei*-construction. Third, we will examine what kind of errors children make in comprehending and producing *ba* and *bei* sentences and discuss why such errors may occur. Together, our results will present a comprehensive picture of how knowledge of sentence structures develops in Mandarin-speaking preschoolers.

## 2. Materials and methods

We conducted a picture selection task (Leonard et al., 2013; Armon-Lotem et al., 2016; Zeng et al., 2016, among many others) to evaluate Mandarin-speaking children’s comprehension of active sentences with the canonical SVO word order, active sentences with a non-canonical S-*ba-*O-V word order, and passive sentences with a non-canonical O-*bei*-S-V word order. We conducted a structural priming task (Messenger et al., 2012a,b; Hao and Chondrogianni, 2021, among many others) to evaluate Mandarin-speaking children’s production of the three types of sentences.

### 2.1. Participants

One hundred and eighty Mandarin-speaking children participated in the two tasks. The participants fell into three age groups: 4-year-olds (*N* = 68, Female = 37, Male = 31; 3;6–4;5, *M* = 4;0), 5-year-olds (*N* = 66, Female = 38, Male = 28; 4;6–5;5, *M* = 5;0), and 6-year-olds (*N* = 46, Female = 24, Male =22; 5;6–6;5, *M* = 5;10). Children were recruited from preschools in Shanghai and Nanjing, China. A consent form was signed by the children’s parents. Children could stop and withdraw from the study at any point they did not want to continue. Data from an additional group of nine children were collected but excluded from analyses because they finished less than two-thirds of the questions. The order of the two tasks was counterbalanced across children.

### 2.2. Stimuli

#### 2.2.1. Sentences and pictures for the comprehension task

For the comprehension task, we created six test sentences for each sentence type, all of which were recorded by a female speaker of Mandarin using a slow conversational speech rate. To increase the variety of the stimuli, among the six sentences of one sentence type, three sentences were shorter with bare nouns and three sentences were longer with the nouns preceded by a color modifier (see examples of two test sentences in (7)). Two additional passive sentences were created for practice (see Supplementary material 1 for a full list of sentences). The test sentences for SVO and *ba*-constructions described the same events (i.e., the noun phrases and the verbs were the same). All bare nouns were common disyllabic words denoting animals familiar to children. All color modifiers had a disyllabic color term *X-se* “X-color” followed by the attributive marker *de* (Li and Thompson, 1981; Yip and Rimmington, 2004). Participants took a pretest color recognition task in which nine colors were shown in a 3*3 grid on the screen (see Supplementary material 2 for the stimuli). Upon hearing a color term from the experimenter, participants were requested to pick out the matched picture. All participants succeeded in identifying all of the nine colors, among which six were used in the task. All verbs were monosyllabic words denoting an action that involved an agent and a patient. According to the Communicative Development Inventory (CDI) for Mandarin Chinese (Hao et al., 2008), apart from two verbs *ya* “press” and *bang* “tie,” all of the words used in the test sentences were already produced by a majority of children at the age of 2;6 (*M* = 83.2%, *SD* = 11.2%). As for the two verbs *ya* “press” and *bang* “tie,” they also appeared in the following production task and children had no problem using these two words. We also considered the semantic connotation of *bei*-construction when choosing verbs for our test sentences. Specifically, the patient underwent some negative influence in the events denoted by verbs such as *bang* “tie.” Therefore, the contexts for using *bei* sentences were appropriate. We had similar considerations for the design of prime sentences in the following production task.

(7) a. shorter trial:

wuguibeixiaomaobang-zhe.

turtleBEIcattie-PROG.

‘The turtle is tied by the cat.’

b. longer trial:

hongse dewuguibeiheisedexiaomao bang-zhe.

redPARTturtleBEIblackPARTcattie-PROG.

‘The red turtle is tied by the black cat.’

**Figure 1:**
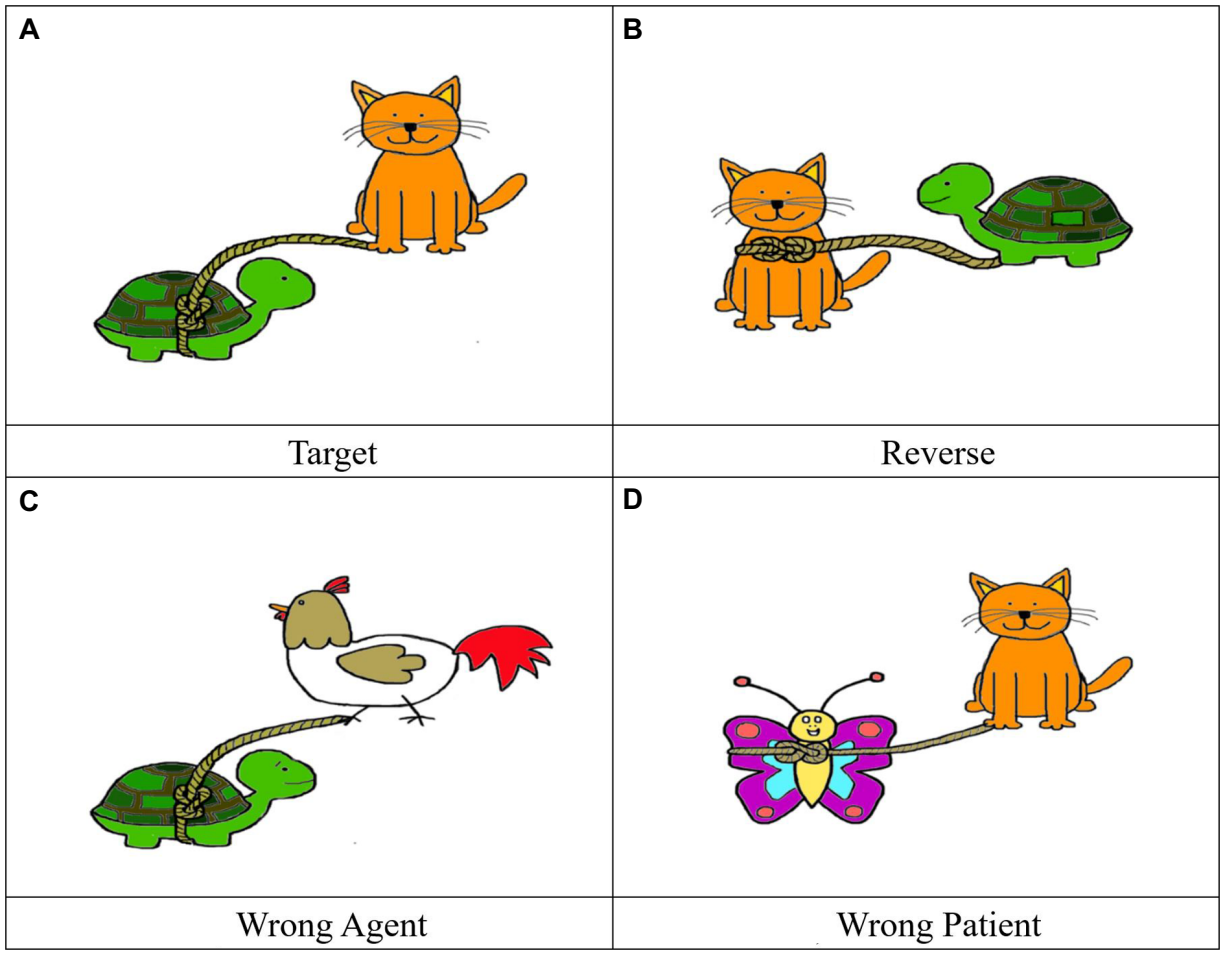
Pictures for test sentence (7a) “The turtle is tied by the cat.”

**Figure 2:**
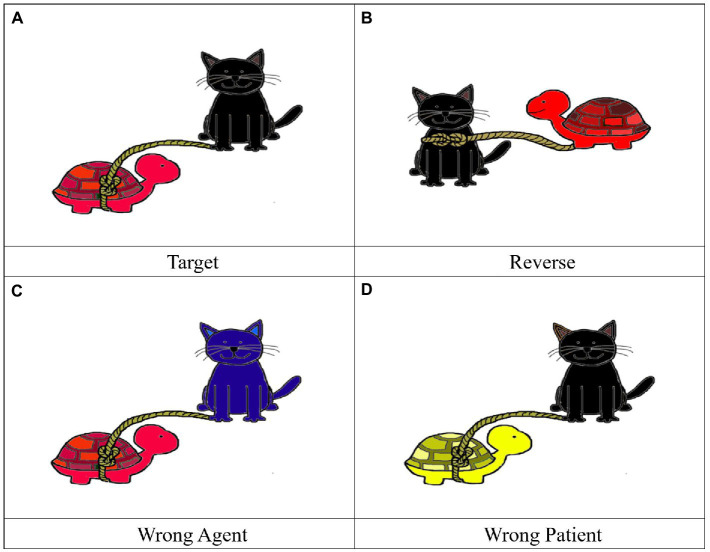
Pictures for test sentence (7b) “The red turtle is tied by the black cat.”

Four pictures including one target and three distractors were designed for each test sentence. For shorter test sentences, the Target picture matched the meaning of the sentence. The Reverse picture had the animals described in the sentence but the thematic relation between the agent and the patient was reverse. In the Wrong Agent picture, the agent animal did not match the test sentence. In the Wrong Patient picture, the patient animal did not match the test sentence. Figure 1 shows the four pictures for (7a). For longer test sentences, the Target picture matched the meaning of the sentence; particularly, the colors of both animals matched the description. In the Reverse picture, both animals and their colors matched the test sentence, but the thematic relation was reverse. In the Wrong Agent picture, the color of the agent animal did not match the test sentence. In the Wrong Patient picture, the color of the patient animal did not match the test sentence Figure 2 shows the four pictures for (7b). The four pictures appeared on a 2*2 split screen with the location of the target and the distractors randomized.

#### 2.2.2. Sentences and pictures for the production task

For the production task, we created eight prime sentences for each sentence type, among which two were used for practice and six were used for testing. Each prime sentence was paired with a target sentence, and the paired prime and target sentences differed only in the second noun phrase as shown in (8) (see Supplementary material 3 for a full list of sentences). We made paired prime and target sentences differ minimally in only one noun phrase because our pilot results showed that young children before 4 years of age had great difficulties producing the target sentence when it differed from the prime sentence in more than one component. The sentences for SVO and *ba*-constructions described the same events (i.e., the noun phrases and the verbs were the same). All bare nouns were di- or tri-syllabic words denoting either common animals or persons familiar to children. All verbs were monosyllabic words denoting an action that involved an agent and a patient. According to the CDI for Mandarin Chinese (Hao et al., 2008), a majority of the words used in this task were already produced by children at the age of 2 years and a half (*M* = 80.8%, *SD* = 0.180). Our pilot results further showed that children had no problems with the words that were not included in the CDI list.

(8) a. youdiyuanti-le **hushi**. (prime sentence)

mailman kick-PFV nurse.

‘The mailman kicked the nurse.’

b. youdiyuan ti-le **jingcha**. (target sentence).

mailman kick-PFV policeman.

‘The mailman kicked the policeman.’

Trials of the same sentence types were blocked. We designed three testing orders “SVO-*bei*-*ba*,” “*ba-*SVO*-bei*,” “SVO-*ba*-*bei*.” Children were randomly assigned to the three orders and the effect of order will be examined in data analysis. Interspersed among the three blocks of target sentence types were trials of other sentence types such as relative clause and pivot construction.

**Figure 3:**
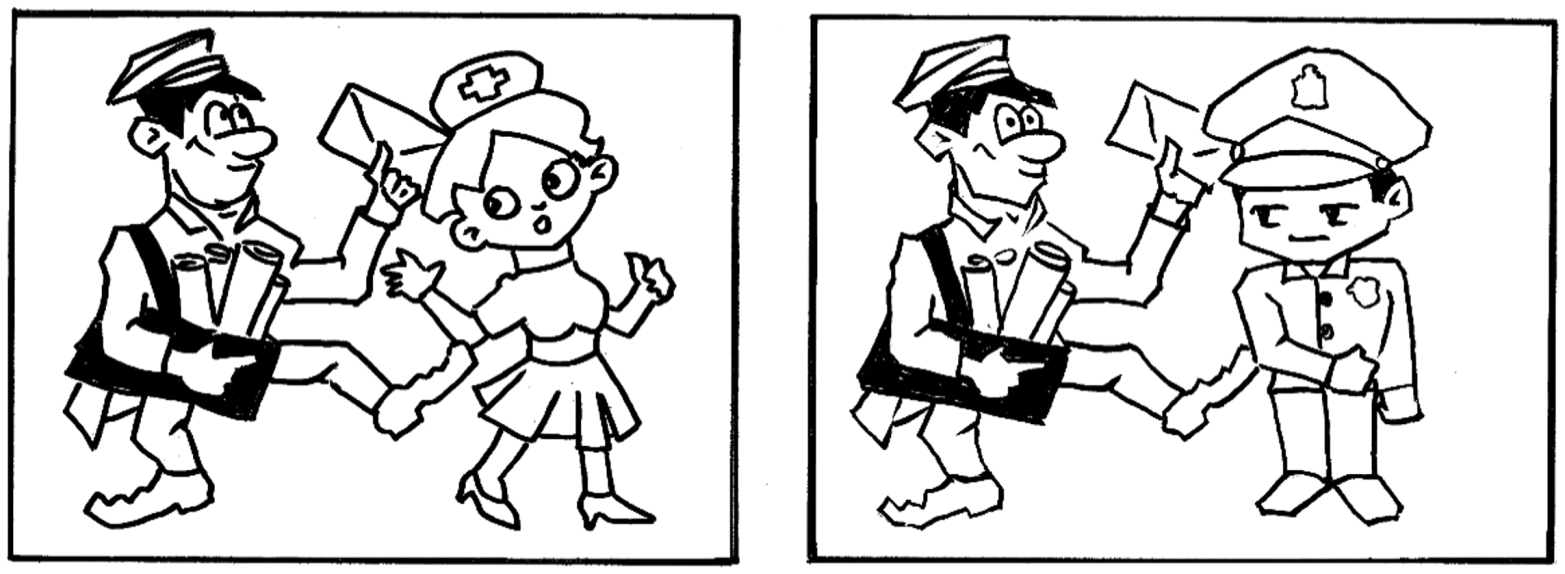
Prime (on the left) and target (on the right) pictures for sentences in (8).

A prime picture and a target picture were created for each prime and target sentence, respectively. Figure 3 shows the pictures for sentences in (8). The two pictures appeared side by side on the screen. The prime picture was always on the left, while the target picture was always on the right.

### 2.3. Procedure

#### 2.3.1. Procedure of the comprehension task

In the comprehension task, children were requested to listen to a recorded test sentence and then point to the picture which best illustrated what they heard. They first had two trials for practice with feedback provided by the experimenter. Afterward, they went through the 18 test sentences in a randomized order. The task reported in the present paper was part of a larger study. The full testing list was composed of 27 sentences. Nine sentences contained constructions (relative clause and pivot construction) not relevant to the present study and were intermixed with the 18 test sentences.

The test sentences were played and the pictures were presented on a laptop using the E-prime software. When children chose a picture, they pushed the corresponding button. Thus, children’s responses were automatically recorded by the software.

#### 2.3.2. Procedure of the production task

In the sentence production task, children saw two pictures along with the experimenter. They were requested to listen to the experimenter talking about what happened in the left picture. Then, their task was to describe what happened in the right picture. In each trial, the experimenter first pointed to each character and named them (e.g., “This is a mailman and this is a nurse. This is a mailman and this is a policeman.”) to ensure that children had no problem with expressing the noun phrase. Then, the experimenter described the left picture with the prime sentence, and afterward asked children what happened in the right picture. In practice trials, if children could not produce the target sentence, the experimenter modeled the target sentence and asked children to repeat it. In test trials, the experimenter only said the prime sentence once. If children asked about the characters, the experimenter reminded them about the noun phrases. Children’s production throughout the task was audio-recorded.

## 3. Results

### 3.1. Results of the comprehension task

#### 3.1.1. Coding

We coded a response as correct when participants chose the Target picture. When participants did not choose the Target picture, the response was coded as an error. Errors were classified into three types: Reverse, Wrong Agent, and Wrong Patient based on which distractor was chosen.

#### 3.1.2. Accuracy

**Figure 4:**
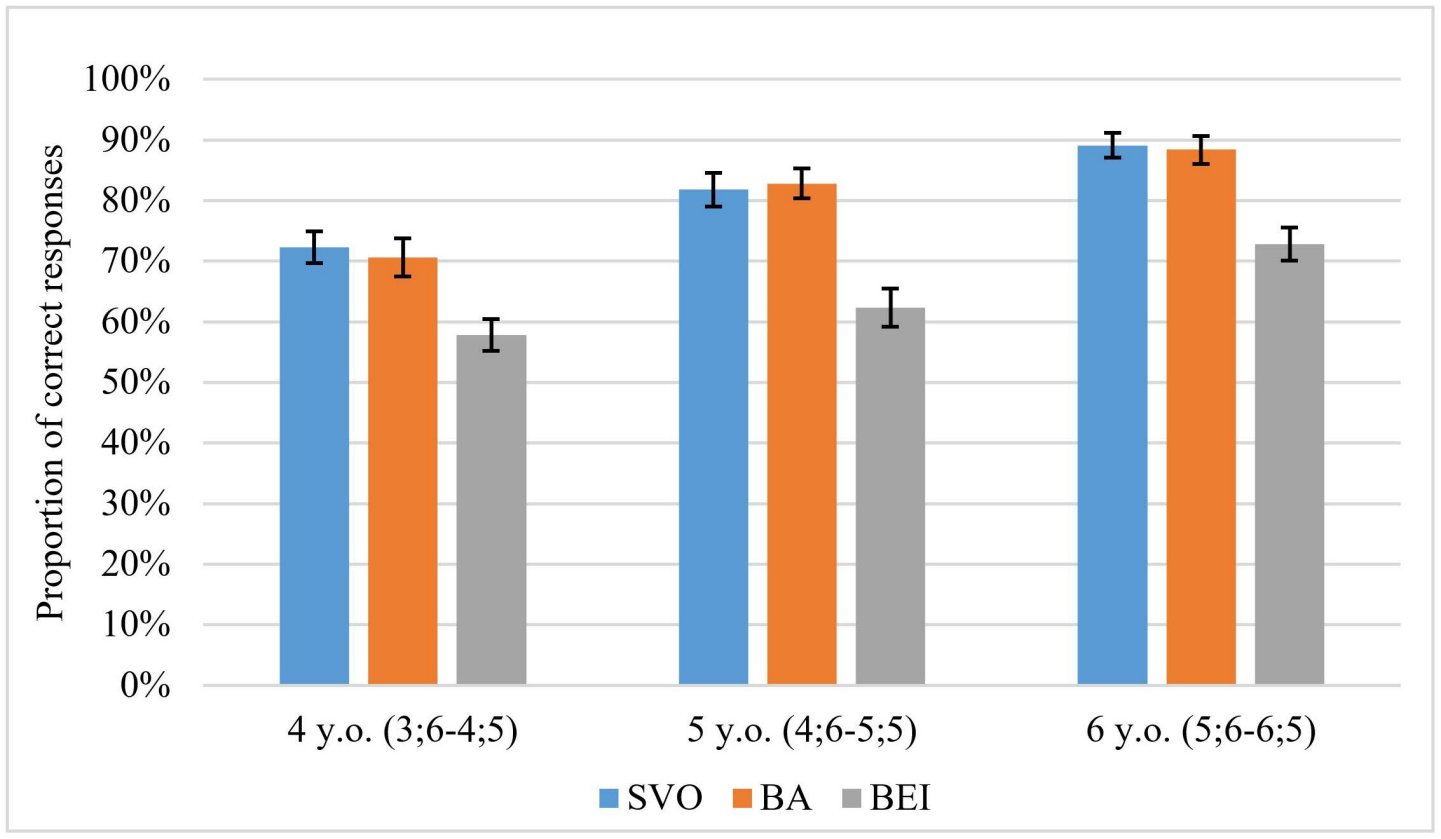
Proportion of correct responses in sentence comprehension. Error bars represent ±SEM.

The descriptive results in Figure 4 show that the proportion of correct responses increased with age. Children comprehended the passive *bei*-construction less accurately than SVO or *ba*-construction.

The binary accuracy data were analyzed using multi-level mixed logit modeling with crossed random intercepts for Subjects and Items (Baayen, 2008; Baayen et al., 2008; Barr, 2008). Based on the guidelines in Barr et al. (2013, p. 275) and follow-up suggestions in Barr (2013, p. 1), we included random slopes for the two within-subjects factors Sentence Type and Sentence Length when building models incrementally. However, models that included a random slope did not converge. Therefore, we kept only random intercepts in our final model. The same treatment of random slopes can be found in other psycholinguistic work (e.g., Kuhn et al., 2021; Lee and Kaiser, 2021). All models were fit using *glmer* function of the *lme4* package in R Project for Statistical Computing (R-Core-Team, 2012).

Since our SVO and *ba*-construction trials involved the same verbs and noun phrases in the test sentences, as well as the same picture stimuli, we first checked whether the order between trials of the two sentence types affected children’s performance. Overall, trials of *ba*-construction appeared 49.3% of the time before their SVO counterparts (i.e., the SVO sentence that had the same verb and noun phrase as the *ba* sentence) and 50.7% of the time after their SVO counterparts. Children’s accuracy of comprehending either sentence type did not differ between the two orders (all *ps* > 0.50). Therefore, the order between trials of SVO and *ba*-construction was not considered in further analysis.

We examined the fixed effects of Age Group (4 y.o. vs. 5 y.o. vs. 6 y.o.), Sentence Type (SVO vs. *ba*-construction vs. *bei*-construction), and Sentence Length (Short vs. Long) as well as their interactions. The fixed effect of Age Group was analyzed with planned comparisons using simple contrast coding (c_1_: 0.66, −0.33, −0.33, c_2_: −0.33, −0.33, 0.66), which set the 5-year-old group as the reference group. Sentence Type was also analyzed with simple contrast coding (c1: −0.33, 0.66, −0.33, c2: −0.33, −0.33, 0.66), which compared comprehension of *ba-*construction and that of *bei-*construction to that of SVO (default word order in Mandarin). The fixed effect of Sentence Length was coded with centered contrast (−0.5, 0.5) and thus, the beta estimate could correctly represent the difference between short and long Sentences. We built up our logit model in a bottom-up fashion: factors of interest (Age Group, Sentence Type, and Sentence Length), a non-theoretically driven predictor Gender, and their interactions were added incrementally to the model to see whether the model fit was improved. Model fit was assessed by Chi-square tests on the log-likelihood values of competing models with three indices, AIC, BIC, and logLik. Predictors or interactions did not reliably improve model fit were excluded from further analysis. The same strategy of model selection was applied to the following analyses. Table 1 reports the final model with parameter estimates of fixed effects.

**Table 1:** Fixed effect estimates for multi-level model of sentence comprehension.

Effect	Estimate	SE	*z*-value
(Intercept)	1.39	0.11	13.00***
Sentence type (SVO vs. *ba*)	−0.05	0.20	−0.23
Sentence type (SVO vs. *bei*)	−1.02	0.20	−5.96***
Sentence length (Short vs. Long)	−0.60	0.16	−3.70***
Age group (5 y.o. vs. 4 y.o.)	−0.54	0.18	−2.98**
Age group (5 y.o. vs. 6 y.o.)	0.55	0.21	2.65**

Formula in R: ACC ~ 1 + SentenceType + AgeGroup + (1 | ID) + (1 | Item).

*p < 0.05, **p < 0.01, ***p < 0.001.

The statistical analysis confirmed the descriptive results. We detected a significant effect of Sentence Type. Specifically, children’s comprehension of *bei*-construction was worse compared to SVO sentences (*z* = −6.96, *p* < 0.001), but there was no significant difference between children’s comprehension of *ba*-construction and SVO sentences (*z* = −0.23, *p* > 0.250). There was also a significant effect of Sentence Length such that children were better in comprehending shorter sentences than the longer ones (*z* = −3.70, *p* < 0.001). Four-year-olds showed worse performance compared with 5-year-olds (*z* = −2.98, *p* = 0.003), while 6-year-olds showed better performance than 5-year-olds (*z* = 2.65, *p* = 0.008). There was a clear development in children’s grammar across age.

We further looked into the difference between the three age groups by counting the number of passers in each age group. For each sentence type, our task had six trials; the probability of choosing the target picture at random was 25%. Based on the binomial distribution, it was extremely unlikely to give the correct response four times out of the six trials by chance (*p* = 0.03). Thus, we considered a participant who gave at least four correct responses among the six trials of a sentence type as a passer in comprehension of that sentence type. Results are shown in Table 2 below.

**Table 2:** Percentages (%) and numbers of participants who performed above chance in comprehending the three sentence types.

Age Group	SVO		*ba-*construction		*bei-*construction	
	%	*N*	%	*N*	%	*N*
4 y.o. (3;6–4;5)	72.06%	49/68	70.59%	48/68	50.00%	34/68
5 y.o. (4;6–5;5)	84.85%	56/66	87.88%	58/66	62.12%	41/66
6 y.o. (5;6–6;5)	97.83%	45/46	93.48%	43/46	78.26%	36/46

In all age groups, there were significantly more children whose comprehension of SVO and *ba*-construction was above chance level compared to comprehension of *bei*-construction (4 y.o.: *Cochran’s Q* = 11.11, *df* = 2, *p* = 0.004; 5 y.o.: *Cochran’s Q* = 18.50, *df* = 2, *p* < 0.001; 6 y.o.: *Cochran’s Q* = 10.31, *df* = 2, *p* = 0.006). This result indicates that the comprehension of passives was delayed.

#### 3.1.3. Error analysis

Children could make three types of errors: Reverse, Wrong Agent, and Wrong patient. Tables 3–5 illustrate the distribution of errors when children failed to choose the correct picture matched with the test sentence. Across the three sentence types, Reverse errors were more common compared to Wrong Agent and Wrong Patient errors.

**Table 3:** Percentages (%) and counts (*N*) of errors in comprehension of SVO sentences.

Age group	Reverse		Wrong agent		Wrong patient	
	%	N	%	N	%	N
4 y.o. (3;6–4;5)	17.89%	73/408	5.15%	21/408	4.66%	19/408
5 y.o. (4;6–5;5)	11.87%	47/396	2.78%	11/396	3.54%	14/396
6 y.o. (5;6–6;5)	6.52%	18/276	2.17%	6/276	2.17%	6/276

**Table 4:** Percentages (%) and counts (*N*) of errors in comprehension of ba-construction.

Age group	Reverse		Wrong agent		Wrong patient	
	%	*N*	%	*N*	%	*N*
4 y.o. (3;6–4;5)	17.40%	71/408	4.90%	20/408	7.11%	29/408
5 y.o. (4;6–5;5)	12.12%	48/396	3.03%	12/396	2.02%	8/396
6 y.o. (5;6–6;5)	6.16%	17/276	2.90%	8/276	2.90%	8/276

**Table 5:** Percentages (%) and counts (*N*) of errors in comprehension of bei-construction.

Age group	Reverse		Wrong agent		Wrong patient	
	%	*N*	%	*N*	%	*N*
4 y.o. (3;6–4;5)	30.15%	123/408	6.13%	25/408	5.88%	24/408
5 y.o. (4;6–5;5)	27.53%	109/396	5.56%	22/396	4.55%	18/396
6 y.o. (5;6–6;5)	22.46%	62/276	2.17%	6/276	2.54%	7/276

Since we were interested in the distribution of different types of errors in each sentence type, the count data of each age group were separately further fit into a generalized linear mixed model with a Poisson distribution and log link. The fixed effect of Error Type was analyzed with planned comparisons using simple contrast coding (c1: −0.33, 0.66, −0.33, c2: −0.33, −0.33, 0.66), which set the Reverse errors as the reference group. The crossed random intercept was provided for Subject. For all age groups, children made more Reverse errors in SVO comprehension than Wrong Agent and Wrong Patient errors (all *ps* < 0.05). A similar pattern was found in comprehension of *ba*-constructions. In both 4-year-old and 5-year-old groups, Reverse errors were more prevalent compared to the other two error types (all *ps* < 0.001). In the oldest group, the difference between Reverse errors and the other two types of errors approached significance (Reverse vs. Wrong Agent: *z* = −1.76, *p* = 0.08; Reverse vs. Wrong Patient: *z* = −1.76, *p* = 0.08). As for the *bei*-construction, Reverse errors were more frequent compared with the other two types of errors (all *ps* < 0.001) in all age groups.

#### 3.1.4. A summary of the comprehension results

Our results illustrate a clear developmental pattern where children’s comprehension of the three sentence types showed a significant improvement between 3;6 and 6;5. As in previous studies (e.g., Chang, 1986; Liu and Ning, 2009; Zeng et al., 2016), we detected delayed acquisition of the passive *bei*-construction. By contrast, though *ba*-construction also involves a non-canonical word order, it did not cause more difficulties for children compared to the canonical SVO word order. This pattern cannot be explained under a usage-based account given the lower frequency of *ba* sentences than SVO sentences; it supports the current syntactic account—the formation of *bei*-construction requires an operator movement out of the vP phase (Huang, 1999), which seems not to be accessible to young children.

When children failed to map the meaning of a sentence with the correct picture, they in most cases could still pick out the correct animals denoted by the noun phrases in the sentence. but they ignored the thematic relationship between the animals. Interestingly, this type of errors was the most common across the board, regardless of sentence types. It suggests that some extra-linguistic factors could play a role in children’s performance. Specifically, to succeed in the task, children had to maintain their attention, hold the test sentences in memory, and suppress the tendency to select the Reverse picture with correct animals but a wrong thematic relation. Future research needs to take children’s cognitive abilities such as working memory and executive functioning into consideration.

### 3.2. Results of the production task

#### 3.2.1. Reliability

Children’s responses were transcribed from the audio recordings by three research assistants who are native speakers of Mandarin. To ensure that the responses were transcribed accurately, we randomly chose 10% of the recordings and asked an additional listener to transcribe. The reliability of transcription measured as the proportion of identical transcriptions (by the additional listener and by the RAs) on a word-by-word basis was 95.4%. Discrepancies (mainly in transcriptions of unintelligible utterances by the youngest group) were resolved by the additional listener and the RAs through discussion.

#### 3.2.2. Coding

We coded a response as correct when it satisfied the following requirements. First, the sentence type was the same as the prime sentence. For production of the *bei*-construction, we allowed using *gei* “give,” *jiao* “let,” and *rang* “allow” instead of *bei* since all these verbs are alternative morphemes that mark passives. Second, the response was a complete sentence. Specifically, we only allowed the omission of subject since Mandarin is a *pro*-drop language. Missing the target noun phrase (i.e., the object noun phrase in SVO sentences, the noun phrase after *ba* in *ba*-construction, and the noun phrase after *bei* in *bei*-construction), the verb, or the aspect marker was regarded as an incomplete sentence. We allowed participants to give slightly different NPs (e.g., a response like *baobao tian-le shushu* “the baby licked the uncle” was coded as correct though the target NP was *baba* “dad” instead of *shushu* “uncle”). Third, the word order was correct such that the thematic relation between the agent and the patient was matched with the picture.

All other responses were coded as errors, which were divided into five types: Incomplete Sentence, Correct Form but Wrong Meaning, Wrong Form but Correct Meaning, Wrong Form and Wrong Meaning, and Other. Incomplete Sentence referred to responses that omitted any of the above-mentioned required elements but included at least one of the required elements. Correct Form but Wrong Meaning meant that the response had the same sentence type as the prime sentence, but the target noun phrase appeared in the wrong place such that the thematic relation was reverse. Wrong Form but Correct Meaning meant that the participant did not use the same sentence type as the prime sentence while the meaning was matched with the picture. Wrong Form and Wrong Meaning meant that the sentence type was not the same as the prime sentence, meanwhile the thematic relation was reverse. The Other category included responses that did not belong to the first four categories as well as unintelligible responses. Table 6 below provides examples illustrating each type of errors.

**Table 6:** Errors in sentence production.

Error type	Target sentence	Child response
Incomplete sentence	youdiyuan ti-le jingcha. Mailman kick-PFV policeman “The mailman kicked the policeman.”	youdiyuan jingcha. Mailman policeman “Mailman, policeman.”
Correct form but wrong meaning	xiaoyang bei xiongmao bang-zhe. Sheep BEI panda tie-PROG “The sheep is tied by the panda.”	xiongmao bei yang bang-zhe. Panda BEI sheep tie-PROG “The panda is tied by the sheep.”
Wrong form but correct meaning	xiaoyang bei xiongmao bang-zhe. Sheep BEI panda tie-PROG “The sheep is tied by the panda.”	xiongmao bang-zhe xiaoyang. Panda tie-PROG sheep “The panda is tying the sheep.”
Wrong form and wrong meaning	baobao ba baba tian-le. baby BA dad lick-PFV “The baby licked dad.”	baobao bei baba tian-le. baby BEI dad lick-PFV “The baby was licked by the dad.”
Other	youdiyuan ti-le jingcha. Mailman kick-PFV policeman “The mailman kicked the policeman.”	you’eryuan. Preschool “Preschool.”

Compared to studies that coded responses based on whether the primed structure was used (e.g., Huttenlocher et al., 2004) or directly categorized responses based on their sentence type (e.g., Hao and Chondrogianni, 2021), our coding and classification of errors could help us better analyze cases when children did not produce the primed sentence type. Particularly, incomplete sentences and sentences that did not correctly describe the target picture were not trivial cases and an analysis of these errors can provide insights into children’s struggles with the meaning vs. form of different sentence types.

#### 3.2.3. Accuracy

**Figure 5:**
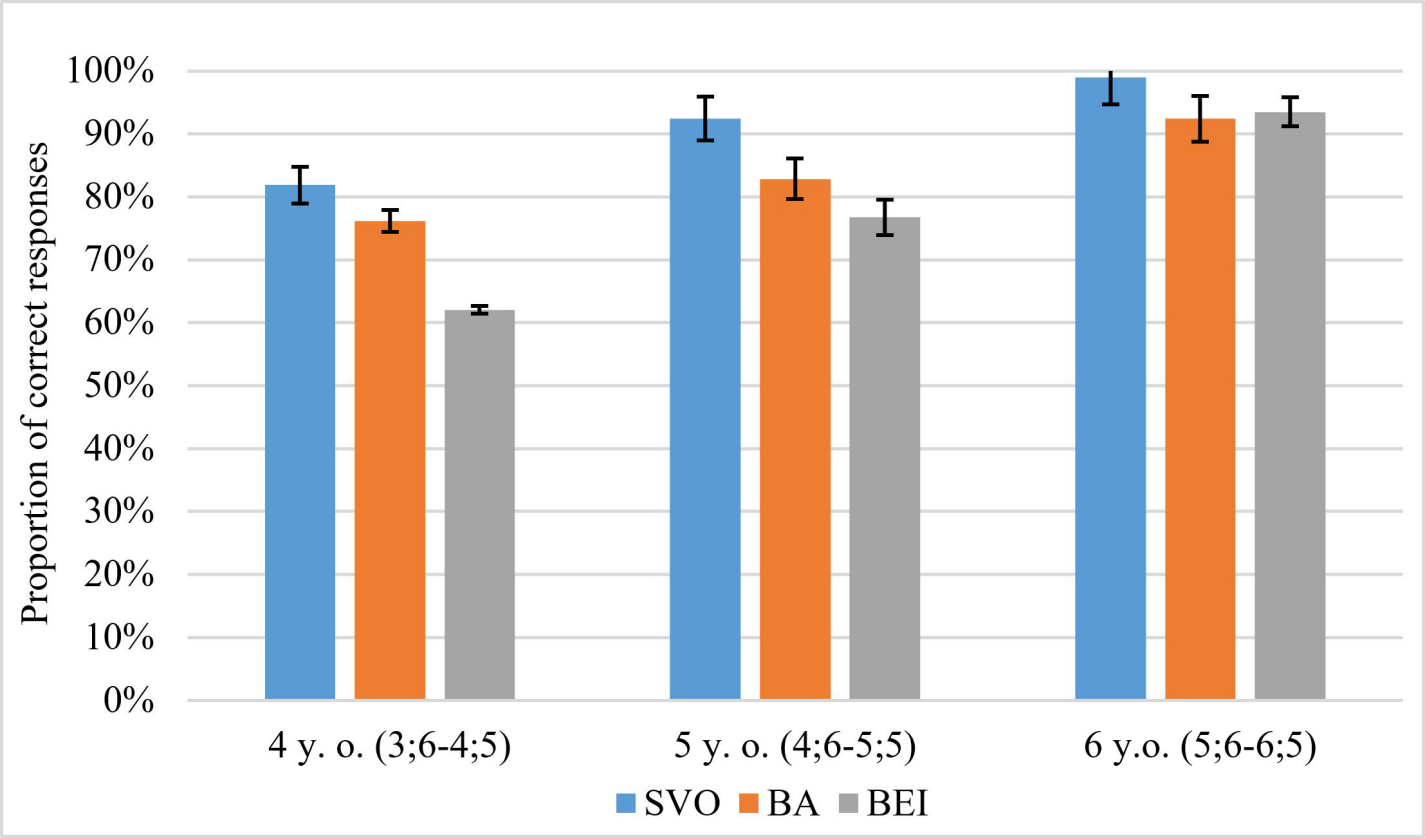
Proportion of correct responses in sentence production. Error bars represent ±SEM.

The descriptive results in Figure 5 show that children produced the most target SVO sentences and the least target passive *bei*-constructions; the performance on *ba*-construction was in between. Older children tended to produce more target sentences than younger children.

We examined the fixed effects of Age Group (4 y.o. vs. 5 y.o. vs. 6 y.o.), Sentence Type (SVO vs. *ba*-construction vs. *bei*-construction), and their interactions. The fixed effect of Age Group was analyzed with planned comparisons using simple contrast coding (c_1_: 0.66, −0.33, −0.33, c_2_: −0.33, −0.33, 0.66), which set the 5-year-old group as the reference group. The fixed effect of Sentence Type was analyzed the same way and coded as (c_1_: −0.33, 0.66, −0.33, c_2_: −0.33, −0.33, 0.66), which compared comprehension of *ba-*construction and of *bei-*construction to that of SVO sentences. We also considered whether the three orders of different sentence types (“SVO-*bei-ba*,” “*ba*-SVO-*bei*,” “SVO-*ba-bei*”) had an effect on children’s performance. The three levels of the Order factor were dummy coded. The binary accuracy data were first submitted to a mixed logit model with Order as a predictor and random intercepts for each Subject and each Item. Other factors of interest and interactions were added incrementally. It turned out that neither Order nor its interaction with other factors significantly improved the model fit (*p* > 0.250), suggesting that trials of one sentence type had little influence on performance in trials of another type. The Order factor was thus excluded from the final model. Table 7 reports parameter estimates of fixed effects.

**Table 7:** Fixed effect estimates for multi-level model of sentence production.

Effect	Estimate	SE	*z*-value
(Intercept)	2.71	0.19	14.21***
Sentence type (SVO vs. *ba*)	−1.28	0.24	−5.27***
Sentence type (SVO vs. *bei*)	−1.68	0.24	−6.90***
Age group (5 y.o. vs. 4 y.o.)	−1.02	0.31	−3.30***
Age group (5 y.o. vs. 6 y.o.)	1.80	0.43	4.21***
SentenceType (SVO vs. *ba*)*AgeGroup (5 y.o. vs. 4 y.o.)	0.79	0.33	2.13*
SentenceType (SVO vs. *ba*)*AgeGroup (5 y.o. vs. 6 y.o.)	−1.10	0.71	−1.55
SentenceType (SVO vs. *bei*)*AgeGroup (5 y.o. vs. 4 y.o.)	0.29	0.32	0.91
SentenceType (SVO vs. *bei*)*AgeGroup (5 y.o. vs. 6 y.o.)	−0.39	0.71	−0.55

Formula in R: ACC ~ 1 + SentenceType + AgeGroup + SentenceType:AgeGroup + (1 | ID) + (1 | Item).

*p < 0.05, **p < 0.01, ***p < 0.001.

The statistical analysis confirmed the descriptive results. There was a significant effect of Sentence Type. Specifically, children produced fewer target sentences of *bei*-construction compared to SVO sentences (*z* = −6.90, *p* < 0.001); they also produced fewer target sentences of *ba*-construction compared to SVO sentences (*z* = −1.28, *p* < 0.001). We also detected the fixed effect of Age Group. Four-year-olds had worse performance compared with 5-year-olds (*z* = −1.02, *p* < 0.001), whose performance was, in turn, worse than 6-year-olds (z = 2.65, *p* < 0.001). There was a clear development in children’s grammar across age. Last, a significant interaction between Sentence Type and Age Group was found (*z* = 2.13, *p* = 0.033): specifically, 4-year-olds did not differ in their production of SVO and *ba*-construction (*odds ratio* = 1.56*, p = 0*.061), but the older age groups had a better performance on SVO sentences compared to *ba-*constructions (5 y.o.: *odds ratio* = 3.12*, p < 0*.001; 6 y.o.: *odds ratio* = 9.14*, p = 0*.002).

Unlike comprehension, there was a difference in children’s production of *ba*-construction and SVO sentences. Therefore, we further compared the production of *bei*-construction and *ba*-construction. Again, the order between the *ba* and *bei* blocks was first examined and excluded since it did not improve the model fit. The accuracy data of *ba-* and *bei*-constructions were submitted to a mixed model with Age Group (4 y.o. vs. 5 y.o. vs. 6 y.o.) and Sentence Type (*ba*-construction vs. *bei*-construction) as predictors. As shown in Table 8, there was a fixed effect of Sentence Type, i.e., children produced more target *ba* sentences than target *bei* sentences (*z* = −2.67, *p* = 0.007). A fixed effect of Age Group was detected: six-year-olds outperformed five-year-olds (*z* = 3.83, *p* < 0.001), who, in turn, outperformed 4-year-olds (*z* = −2.56, *p* = 0.01). Furthermore, there was a significant interaction between the two predictors (*z* = −2.98, *p* = 0.004). To interpret the interaction, we used *emmeans* package in R (Lenth, 2020) to compute pairwise comparisons to examine the effect of Sentence Type in each age group. It turned out that only the youngest group had better performance on *ba*-construction than *bei*-construction (*odds ratio* = 2.51, *p* < 0.001); 5-year-olds and 6-year-olds did not differ between the two sentence types (5 y.o.: *odds ratio* = 1.66, *p* = 0.140; 6 y.o.: *odds ratio* = 0.82, *p* > 0.250).

**Table 8:** Fixed effect estimates for multi-level model of the production of ba- and bei-constructions.

Effect	Estimate	SE	*z*-value
(Intercept)	2.27	0.17	13.13***
Sentence type (*ba* vs. *bei*)	−0.41	0.15	−2.67**
Age group (5 y.o. vs. 4 y.o.)	−0.84	0.33	−2.57*
Age group (5 y.o. vs. 6 y.o.)	1.59	0.42	3.83***
SentenceType (*ba* vs. *bei*)*AgeGroup (5 y.o. vs. 4 y.o.)	−0.71	0.42	−2.98**
SentenceType (*ba* vs. *bei*)*AgeGroup (5 y.o. vs. 6 y.o.)	0. 41	0.28	1.48

Formula in R: ACC ~ 1 + SentenceType + AgeGroup + SentenceType:AgeGroup + (1 | ID) + (1 | Item) + (1 + SentenceType | ID).

*p < 0.05, **p < 0.01, ***p < 0.001.

#### 3.2.4. Error analysis

**Figure 6:**
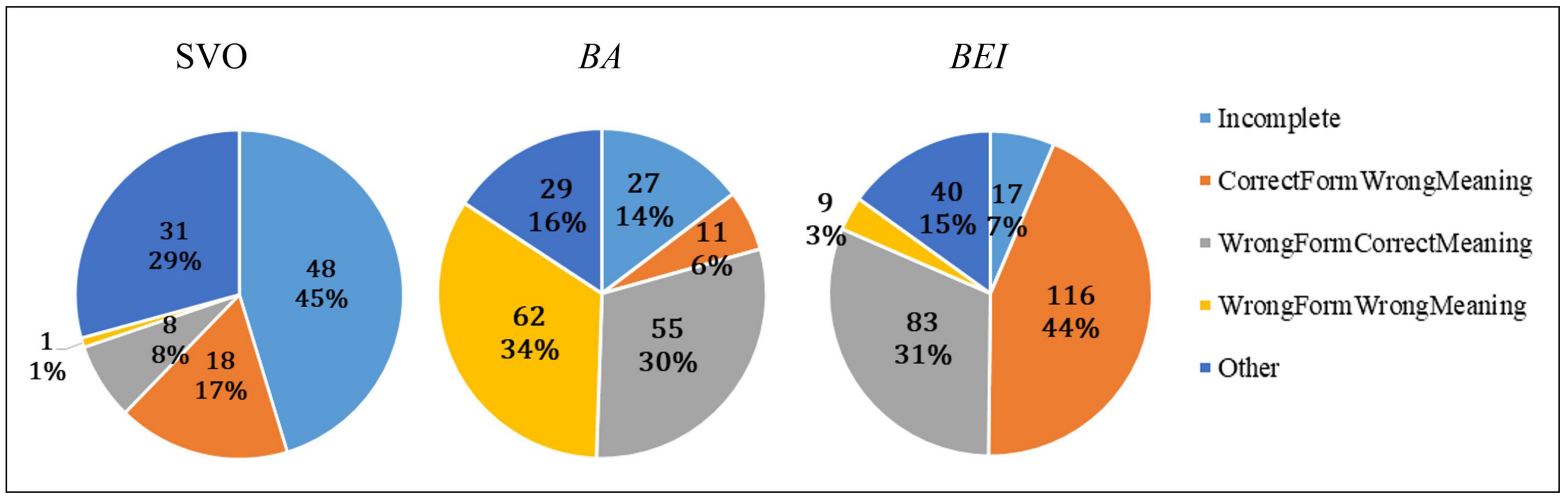
The distribution of different errors across the three sentence types.

Five types of errors were detected: Incomplete Sentence, Correct Form but Wrong Meaning, Wrong Form but Correct Meaning, Wrong Form and Wrong Meaning, and Other. Figure 6 shows the counts and proportions of different types of errors. We collapsed data of the three age groups since some types of errors were very rare and the performance of the oldest group was almost at ceiling. From the first sight, the distribution of different types of errors varied greatly across the three sentence types. We further analyzed errors for each sentence type separately.

The counts of errors were submitted to a generalized linear mixed model with a Poisson distribution. The fixed effect of Error Type was analyzed with planned comparisons using deviation coding that compared the count of one type of errors to the overall mean of errors. The crossed random intercept was provided for Subject. By including the variation in subjects, the model evaluates whether a type of errors was scattered across many children or was found in just a few children. The random slope was also included since it significantly improved the model fit. This suggests that different subjects have different error distributions.

For SVO sentences, the most frequent errors were of the Incomplete-Sentence type, which were significantly more common than average (z = 5.70, *p* < 0.001). Among the 48 incomplete responses, 27 included the target noun phrase only while the verb and aspect marker were missing (see the first example in Table 6); 16 had the verb and aspect marker but the target noun phrase was missing; 5 had the verb and the target noun phrase, but the aspect marker was missing.

As for *ba*-construction, the most frequent errors were classified as Wrong Form and Wrong Meaning. Specifically, *ba* was replaced by the passive morpheme *bei* or *gei*, resulting in a non-target form and a reverse thematic relationship (see the fourth example in Table 6). However, such errors were not significantly more common than average (*z* = −1.53, *p* = 0.125). In fact, this type of errors was uncommon, as indicated by the negative Z value. Such errors were found in only 23 out of 180 participants, among which 13 children replaced *ba* with a passive morpheme in 1 or 2 trials and 10 children did so in at least 3 trials. In other words, a small group of children used the passive morpheme rather than *ba*. This was not caused by a perseveration from completing the block of *bei*-construction first, since 15 out of the 23 participants encountered the *ba* block first. It was not due to potential influence from dialects other than Mandarin either—9 out of 23 children had a parent speaking a southern dialect such as Shanghai dialect which does not allow replacement of *ba* with other morphemes, 13 children had both parents speaking Mandarin only, and one child had parents speaking a Northeast dialect. At present, we could not provide an explanation for this pattern. The second most frequent errors were of the Wrong-Form-but-Correct-Meaning type, which were more common than average (*z* = 4.39, *p* < 0.001). Among the 55 instances of such errors, 49 were SVO sentences and 6 were passive sentences with a passive morpheme *bei* or *gei*.

As for the production of *bei*-construction, the most frequent errors belonged to the type of Correct Form but Wrong Meaning. Such errors were more common than average (*z* = 6.16, *p* < 0.001). Although the passive structure was the same as the prime sentence, the two noun phrases appeared in a wrong order, thus the agent-patient relationship was reverse (see the second example in Table 6). Similar to *ba*-construction, the second most frequent errors involved Wrong Form but Correct Meaning, which were more common than average (*z* = 4.82, *p* < 0.001). Among the 83 instances of such errors, 54 were SVO sentences and 29 were *ba*-constructions.

#### 3.2.5. A summary of the production results

Similar to the results of sentence comprehension, children’s production showed significant improvement between 3;6 and 6;5. Performance of the oldest group was nearly at ceiling. However, unlike comprehension, the youngest children produced significantly less target *ba*-constructions than SVO sentences. This suggests that although non-canonical word order does not necessarily lead to delayed comprehension, it may cause some difficulties in early production. In addition, children had different problems with producing *ba*- and *bei*-construction. Unlike earlier reports from naturalistic studies that 4-year-olds were productive and almost adult-like in using the *ba*-construction, we found considerable instances where children switched to the canonical SVO word order rather than producing *ba* sentences. As for *bei*-construction, our results showed that though children could be primed on the passive structure, they made many errors in the agent-patient relationship and seemed to confuse the structure with active sentences.

## 4. Discussion

Our results illustrated clear development of Mandarin-speaking children’s syntactic knowledge from 3;6 to 6;5. Specifically, as children grew older, they became better at understanding and producing non-canonical *ba* and *bei* sentences. Both non-canonical word orders caused some difficulties for children compared to the canonical SVO sentences. In comprehension, children made more errors in understanding *bei* sentences though their performance on *ba*-construction and SVO sentences did not differ. In production, children achieved high accuracy in producing target SVO sentences, but they produced less target *ba* sentences and even less target *bei* sentences.

### 4.1. Early difficulties with the passive *Bei*-construction

Our study showed delayed acquisition of the passive *bei*-construction—even 6-year-olds made considerable errors in both tasks, and this finding is in line with previous research (e.g., Chang, 1986; Liu and Ning, 2009; Zeng et al., 2016). Furthermore, we identified the major errors in Mandarin-speaking children: they tended to regard passives as active sentences and this resulted in a reverse agent-patient relationship in both comprehension and production. This pattern is consistent with what has been found in English-speaking children (e.g., Pinker et al., 1987; Gordon and Chafetz, 1990; Brooks and Tomasello, 1999; Messenger et al., 2012b; *cf.* Armon-Lotem et al., 2016) and provides evidence for the universality of Wexler’s (2004, 2007) proposal, i.e., movement from the object position out of the vP phase is not available in immature grammar due to a lack of knowledge about defective *v*.

However, there is a challenge for attributing children’s difficulties with *bei*-construction solely to the maturation of grammar. The maturation account would predict more “categorical” performance—before knowledge about defective *v* matures, children should have very poor performance with *bei* sentences. After the knowledge becomes available, there should be a remarkable boost in children’s performance. Our cross-sectional comprehension and production data both show gradual improvement across age groups in *bei*-construction, which poses questions about what actually drives this improvement. In effect, gradual development of a syntactic construction is expected by the usage-based account - as children grow older, they have more exposure to *bei*-sentences and their performance becomes better due to their familiarity with the construction. To tease apart the contribution of syntactic knowledge and construction frequency, future research can compare the acquisition of *bei*-construction with a construction that is equally rare in the input but does not involve long-distance movement out of *v*P. Future studies may also utilize a longitudinal design to better capture at which point children’s knowledge of long-distance movement matures.

### 4.2. Acquisition of *Ba*-construction

Our study also presented a comprehensive picture of the acquisition of *ba*-construction. In comprehension, no difference between *ba* and SVO sentences was detected. This may be taken as direct support for the maturation account which assumes that movement within the vP phase is available from the beginning. However, in the comprehension task, children could take some simpler strategy without processing the sentence structure. For instance, upon hearing the first noun phrase, they quickly associate it with the agent since the first argument of a sentence typically takes an agent role in their language (Yang et al., 2003); then they assign the patient role to the second noun phrase they hear. This possibility would be compatible with predictions from cue-based competition models (e.g., Bates and MacWhinney, 1987, 1989) which proposed that word order is a more reliable cue compared to morphological markers such as the passive *–en* in English (e.g., MacWhinney et al., 1984) and *ba* in Mandarin (Li et al., 1993). To evaluate this alternative explanation, future research may collect online processing data such as eye gaze patterns that can reveal how children arrive at the correct interpretation. Specifically, if children simply map arguments to thematic roles based on their order, then *ba* sentences should be processed at a faster speed since the second noun phrase appears earlier compared to SVO sentences. But if children comprehend sentences by analyzing the syntactic structure, then *ba* sentences might need more time due to the movement operation.

In contrast to comprehension, children’s production of *ba*-construction was worse than SVO sentences. On the one hand, a small group of children have replaced *ba* with a passive morpheme; for 80.65% of these cases, *gei* was used. Though at present we cannot provide an assured explanation to this pattern, historical and dialectal works on *gei* show that this morpheme, originally a ditransitive verb meaning “give,” has undergone grammaticalization and can be used as an agent marker like *bei* or as a patient marker like *ba* at least in Beijing dialect (Xu, 1994; Her, 2006). Further studies are needed to elucidate what children mean in their use of *gei*. On the other hand, children tended to change a *ba* sentence into a canonical SVO sentence. Remember that our paired prime and target sentences differed only in one component (i.e., the object NP in both *ba* and SVO sentences). To successfully produce a target *ba* sentence, children had to retain the *ba* morpheme and the verb in their working memory and insert the new object NP in between. By contrast, producing an SVO sentence without *ba* and a preverbal object was much less demanding. Future research may include some measurement of working memory capacity to better compare children’s production of *ba* and SVO sentences. In addition, the high frequency of SVO sentences may also lead to this pattern but the exact relationship between the frequency of a construction in the input and the likelihood of a child to use the construction remains to be explored.

### 4.3. Comparisons between *Ba, Bei*, and SVO sentences

Comparing between children’s performance on *ba* and *bei* sentences, our study revealed that children were better at comprehending *ba*-construction, which is expected by the maturation account, as well as the usage-based account since *ba* sentences are more frequent in child-directed speech than *bei* sentences. Unlike comprehension, only the youngest group had a better performance in the production of *ba*- than *bei*-construction. The finding that the older children performed comparably on *ba*- and *bei*-production cannot be explained under either the maturation or the usage-based account. It is possible that the production task may have encouraged the use of response strategies among the older children and masked their difficulty with *bei*- compared to *ba*-production—they may have taken the shortcut of simply replacing the second noun phrase in the target sentence without extracting the sentence structure. The literature on structural priming suggests that speakers start with a functional level of representation (i.e., linguistic expressions are encoded in terms of their grammatical functions such as subject, direct object; Garrett, 1980), which is mapped onto the specific structure of the prime sentence either in a separate step (see the two-stage model in Hartsuiker et al., 1999) or within the same stage (Pickering et al., 2002). In our production task, the functional representation was “half established” since the first noun phrase and the verb were exactly the same in paired prime and target sentences. In the future, harder priming tasks [e.g., using different lexical items in the target sentence as in Messenger et al. (2012a) and Hao and Chondrogianni (2021)] should be conducted among older children to better tap into their use of different sentence types. Particularly, performance of 5-year-olds and 6-year-olds is worth further investigation as Messenger et al. (2012a) have found that 6-year-old English-speaking children produced reversed passives, but Hao and Chondrogianni (2021) detected adult-like performance in 5-to-9-year-old Mandarin-speaking children.

Last but not least, our study showed some similarities and differences between children’s comprehension and production of the three sentence types. Performance in older children suggests that overall, the comprehension task was more difficult than the production task. At least two task-related factors may have caused this production advantage. First, each trial of the comprehension task was composed of four colored pictures, which differed minimally from each other in only one aspect. To succeed, children had to differentiate the pictures, and reject three distractors. By contrast, the production task had two black-and-white pictures differing in one figure in each trial. Unlike the comprehension task, the similarities between the two pictures could help children produce the target sentence. Second, the comprehension task used a totally randomized list such that the sentence type tended to vary from trial to trial. Children had to activate different syntactic representations throughout the testing. In comparison, in the production task, trials of the same sentence type were presented in one block. Children did not need to switch to a different syntactic structure before reaching a new block. Due to possible differences in task demands, at present, we cannot specify the relationship between comprehension and production apart from showing a strong positive correlation between children’s performance on both tasks (*Pearson’s r* = 0.490, *p* < 0.001). Future research needs to investigate what are shared and what may differ in language comprehension and language production, which is still a controversial issue (e.g., Chapman and Miller, 1975; Clark and Hecht, 1983; Håkansson and Hansson, 2000; Segaert et al., 2012). In effect, the maturation account is often supported by results from comprehension studies (for a summary of empirical studies, see Crain and Pietroski, 2002), while the usage-based account is discussed more with production data (for a review, see Abbot-Smith and Tomassello, 2016). A better understanding of the relationship may shed light on the debate between the two accounts of language acquisition.

## 5. Conclusion

Our study investigates the acquisition of Mandarin non-canonical word orders through a comprehension and a production task with a large sample of 180 Mandarin-speaking children between 3 and 6 years of age. Our results show that children have more difficulties with the passive *bei*-construction compared to SVO sentences in both comprehension and production, but early problems of *ba*-construction only lie in production. These findings contribute novel information on the development of Mandarin-speaking children’s syntactic knowledge from Age 3 to Age 6. On the one hand, our study provides insights into two major theories of language acquisition which attribute language development to the maturation of grammar or to the exposure to the input, respectively. On the other hand, our data present challenges to existing theories that call for future studies using a longitudinal design and/or online processing techniques.

## Data availability statement

The datasets presented in this study can be found in online repositories. The names of the repository/repositories and accession number(s) can be found below: the Open Science Framework (https://osf.io/7ru43/, the “Acquisition of non-canonical word orders in Mandarin Chinese” project).

## Ethics statement

The studies involving human participants were reviewed and approved by Hong Kong Polytechnic University. Written informed consent to participate in this study was provided by the participants’ legal guardian/next of kin.

## Author contributions

YJ performed data analysis and wrote the first draft of the manuscript. LS was the principal investigator of the whole project on Mandarin preschool children’s language development and reviewed and edited the manuscript. LZ supervised the data collection process. All authors contributed to the study conceptualization and design, and approved the submitted version.

## Funding

This material is based upon work supported by the Beijing Institute of Technology Research Fund Program for Young Scholars (YJ), the National Social Science Fund of China (Grant 21CYY046; YJ), the Pu Dong One Hundred Award (LS), and the Humanities and Social Sciences projects of the Chinese Ministry of Education (Grant 17YJAZH132, LZ and LS).

## Conflict of interest

The authors declare that the research was conducted in the absence of any commercial or financial relationships that could be construed as a potential conflict of interest.

## Publisher’s note

All claims expressed in this article are solely those of the authors and do not necessarily represent those of their affiliated organizations, or those of the publisher, the editors and the reviewers. Any product that may be evaluated in this article, or claim that may be made by its manufacturer, is not guaranteed or endorsed by the publisher.

## Abbreviations

PFV, perfective aspect marker
PROG, progressive aspect marker
PART, particle
BEI, passive morpheme *bei*
BA, disposal marker *ba*
IP, inflection phrase
CP, complementizer phrase
OP, operator
