# Supplementary material for: Acquisition of non-canonical word orders in Mandarin Chinese
Source: Front Psychol. 2023 Apr 17;14:1006148. doi: 10.3389/fpsyg.2023.1006148 (PMC10149943; doi:10.3389/fpsyg.2023.1006148)
Supplement: Supplementary file 1 [file Table_1.DOCX]

Supplementary Material

# Sentences in the comprehension task

## Two sentences for practice.

- 1. xiaoji bei qingwa bao-zhe.

chicken BEI frog hug-PROG

‘The chicken is hugged by the frog.’

- 1. huangse de xiaoji bei hongse de qingwa bao-zhe.

yellow PART chicken BEI red PART frog hug-PROG

‘The yellow chicken is hugged the red frog.’

## 6 SVO sentences

- 1. xiaoji bao-zhe qingwa.

chicken hug-PROG frog

‘The chicken is hugging the frog.’

- 1. huangse de xiaoji bao-zhe hongse de qingwa.

yellow PART chicken hug-PROG red PART frog

‘The yellow chicken is hugging the red frog.’

- 1. daxiang ti-le laoshu.

elephant kick-PFV mouse

‘The elephant kicked the mouse.’

- 1. lanse de daxing ti-le baise de laoshu.

blue PART elephant kick-PFV white PART mouse

‘The blue elephant kicked the white mouse.’

- 1. xiaoniao zhuang-le wugui.

bird hit-PFV turtle

‘The bird hit the turtle.’

- 1. heise de xiaonao zhuang-le huangse de wugui.

black PART bird hit-PFV yellow PART turtle

‘The black bird hit the yellow turtle.’

## 6 sentences of ba-construction

- 1. xiaoji ba qingwa bao-zhe.

chicken BA frog hug-PROG

‘The chicken is hugging the frog.’

- 1. huangse de xiaoji ba hongse de qingwa bao-zhe.

yellow PART chicken BA red PART frog hug-PROG

‘The yellow chicken is hugging the red frog.’

- 1. daxiang ba laoshu ti-le.

elephant BA mouse kick-PFV

‘The elephant kicked the mouse.’

- 1. lanse de daxing ba baise de laoshu ti-le.

blue PART elephant BA white PART mouse kick-PFV

‘The blue elephant kicked the white mouse.’

- 1. xiaoniao ba wugui zhuang-le.

bird BA turtle hit-PFV

‘The bird hit the turtle.’

- 1. heise de xiaonao ba huangse de wugui zhuang-le.

black PART bird BA yellow PART turtle hit-PFV

‘The black bird hit the yellow turtle.’

## 6 sentences of bei-construction

- 1. xiongmao bei tuzi da-le.

panda BEI rabbit beat-PFV

‘The panda was beaten by the rabbit.’

- 1. lvse de xiongmao bei huangse de tuzi da-le.

green PART panda BEI yellow PART rabbit beat-PFV

‘The green panda was beaten by the yellow rabbit.’

- 1. qingwa bei laoshu ya-zhe.

frog BEI mouse press-PROG

‘The frog is pressed by the mouse.’

- 1. baise de qingwa bei hongse de laoshu ya-zhe.

white PART frog BEI red PART mouse press-PROG

‘The white frog is pressed by the red mouse.’

- 1. wugui bei xiaomao bang-zhe.

turtle BEI cat tie-PROG

“The turtle is tied by the cat.”

- 1. hongse de wugui bei heise de xiaomao bang-zhe.

red PART turtle BEI black PART cat tie-PROG

‘The red turtle is tied by the black cat.’

# The picture stimuli in the color recognition task

| 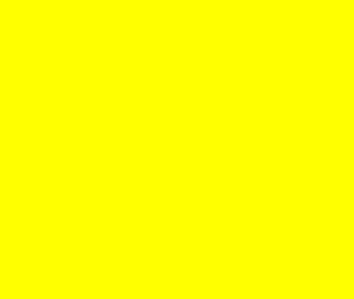 | 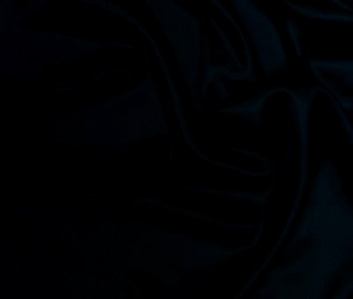 | 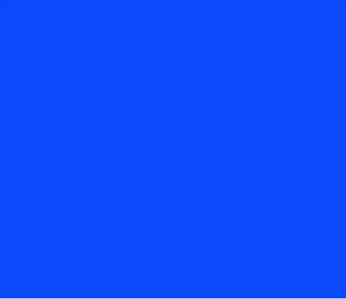 |
| --- | --- | --- |
| 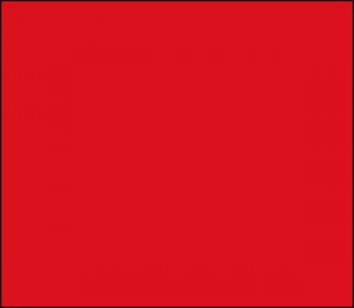 | 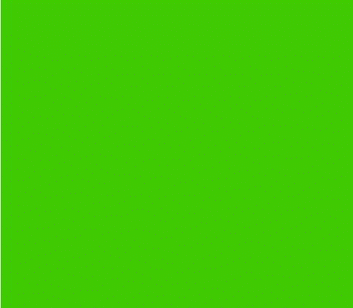 | 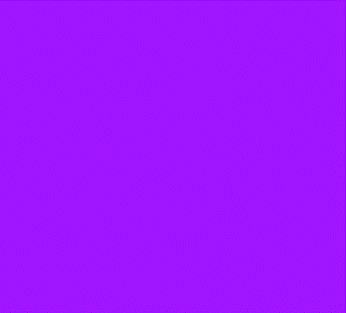 |
| 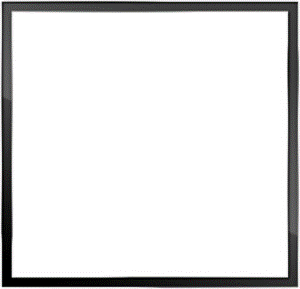 | 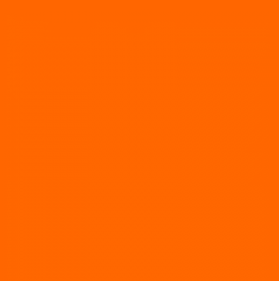 | 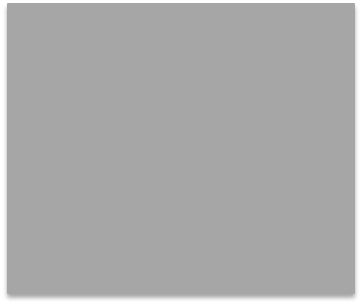 |

**Supplementary Figure 1.** The picture stimuli in the color recognition task

# Sentences in the production task

For each sentence type, the first two sentences are used for practice; the rest six sentences are used for testing.

## SVO sentences

- 1. a. meimei yao-le didi. (prime sentence)

younger sister bite-PFV younger brother

‘Younger sister bit younger brother.’

b. meimei yao-le gege. (target sentence)

younger sister bite-PFV elder brother

‘Younger sister bit elder brother.’

- 1. a. baobao tian-le mama. (prime sentence)

baby lick-PFV mom

‘Baby licked mom.’

b. baobao tian-le baba. (target sentence)

baby lick-PFV dad

‘Baby licked dad.’

- 1. a. youdiyuan ti-le hushi. (prime sentence)

mailman kick-PFV nurse

‘The mailman kicked the nurse.’

b. youdiyuan ti-le jingcha. (target sentence)

mailman kick-PFV policeman

‘The mailman kicked the policeman.’

- 1. a. houzi zhuang-le xiaozhu. (prime sentence)

monkey hit-PFV pig

‘The monkey hit the pig.’

b. houzi zhuang-le laoshu. (target sentence)

monkey hit-PFV mouse

‘The monkey hit the mouse.’

- 1. a. xiaozhu bao-zhe maotouying. (prime sentence)

pig hug-PROG owl

‘The pig is hugging the owl.’

b. xiaozhu bao-zhe xiaogou. (target sentence)

pig hug-PROG dog

‘The pig is hugging the dog.’

- 1. a. xiaolu ya-zhe xiaoniu. (prime sentence)

deer press-PROG calf

‘The deer is pressing the calf.’

b. xiaolu ya-zhe xiaoyang. (target sentence)

deer press-PROG sheep

‘The deer is pressing the sheep.’

- 1. a. mayi bangzhe luotuo. (prime sentence)

ant tie-PROG camel

‘The ant is tying the camel.’

b. mayi bangzhe houzi. (target sentence)

ant tie-PROG monkey

‘The ant is tying the camel.’

- 1. a. xiaoniu la-zhe xiaoyang. (prime sentence)

calf pull-PROG sheep

‘The calf is pulling the sheep.’

b. xiaoniu la-zhe xiaozhu. (target sentence)

calf pull-PROG pig

‘The calf is pulling the pig.’

## *ba*-construction

- 1. a. meimei ba didi yao-le. (prime sentence)

younger sister BA younger brother bite-PFV

‘Younger sister bit younger brother.’

b. meimei ba gege yao-le. (target sentence)

younger sister BA elder brother bite-PFV

‘Younger sister bit elder brother.’

- 1. a. baobao ba mama tian-le. (prime sentence)

baby BA mom lick-PFV

‘Baby licked mom.’

b. baobao ba baba tian-le. (target sentence)

baby BA dad lick-PFV

‘Baby licked dad.’

- 1. a. youdiyuan ba hushi ti-le. (prime sentence)

mailman BA nurse kick-PFV

‘The mailman kicked the nurse.’

b. youdiyuan ba jingcha ti-le. (target sentence)

mailman BA policeman kick-PFV

‘The mailman kicked the policeman.’

- 1. a. houzi ba xiaozhu zhuang-le. (prime sentence)

monkey BA pig hit-PFV

‘The monkey hit the pig.’

b. houzi ba laoshu zhuang-le. (target sentence)

monkey BA mouse hit-PFV

‘The monkey hit the mouse.’

- 1. a. xiaozhu ba maotouying bao-zhe. (prime sentence)

pig BA owl hug-PROG

‘The pig is hugging the owl.’

b. xiaozhu ba xiaogou bao-zhe. (target sentence)

pig BA dog hug-PROG

‘The pig is hugging the dog.’

- 1. a. xiaolu ba xiaoniu ya-zhe. (prime sentence)

deer BA calf press-PROG

‘The deer is pressing the calf.’

b. xiaolu ba xiaoyang ya-zhe. (target sentence)

deer BA sheep press-PROG

‘The deer is pressing the sheep.’

- 1. a. mayi ba luotuo bangzhe. (prime sentence)

ant BA camel tie-PROG

‘The ant is tying the camel.’

b. mayi ba houzi bangzhe. (target sentence)

ant BA monkey tie-PROG

‘The ant is tying the camel.’

- 1. a. xiaoniu ba xiaoyang la-zhe. (prime sentence)

calf BA sheep pull-PROG

‘The calf is pulling the sheep.’

b. xiaoniu ba xiaozhu la-zhe. (target sentence)

calf BA pig pull-PROG

‘The calf is pulling the pig.’

## *Bei*-construction

- 1. a. laohu bei xiaozhu yao-le (prime sentence)

tiger BEI pig bite-PFV

‘The tiger was bitten by the pig.’

b. laohu bei e’yu yao-le (target sentence)

tiger BEI crocodile bite-PFV

‘The tiger was bitten by the crocodile.’

- 1. a. changjinglu bei xiaolu pai-le. (prime sentence)

giraffe BEI deer pat-PFV

‘The giraffe was patted by the deer.’

b. changjinglu bei houzi pai-le. (target sentence)

giraffe bei monkey pat-PFV

‘The giraffe was patted by the monkey.’

- 1. a. xiaoniu bei kongque tui-le. (prime sentence)

calf BEI peacock push-PFV

‘The calf was pushed by the peacock.’

b. xiaoniu bei qi’e tui-le. (target sentence)

calf BEI penguin push-PFV

‘The calf was pushed by the penguin.’

- 1. a. mayi bei xiaoyang zhuang-le. (prime sentence)

ant BEI sheep hit-PFV

‘The ant was hit by the sheep.’

b. mayi bei xiaogou zhuang-le. (target sentence)

ant BEI dog hit-PFV

‘The ant was hit by the dog.’

- 1. a. baobao bei baba bao-zhe. (prime sentence)

baby BEI dad hold-PROG

‘The baby is held by dad.’

b. baobao bei mama bao-zhe. (target sentence)

baby BEI mom hold-PROG

‘The baby is held by mom.’

- 1. a. didi bei shushu tuo-zhe. (prime sentence)

younger brother BEI uncle pull-PROG

‘The younger brother is pulled by uncle.’

b. didi bei a’yi tuo-zhe. (target sentence)

younger brother BEI aunt pull-PROG

‘The younger brother is pulled by aunt.’

- 1. a. xiaoyang bei da huilang bang-zhe. (prime sentence)

sheep BEI big wolf tie-PROG

‘The sheep is tied by the big wolf.’

b. xiaoyang bei xiongmao bang-zhe. (target sentence)

sheep BEI panda tie-PROG

‘The sheep is tied by the panda.’

- 1. a. yeye bei jiejie fu-zhe. (prime sentence)

grandpa BEI elder sister support-PROG

‘Grandpa is supported by elder sister.’

b. yeye bei didi fu-zhe. (target sentence)

grandpa BEI younger brother support-PROG

‘Grandpa is supported by younger brother.’
